# An Asynchronous Low Power and High Performance VLSI Architecture for Viterbi Decoder Implemented with Quasi Delay Insensitive Templates

**DOI:** 10.1155/2015/621012

**Published:** 2015-10-07

**Authors:** T. Kalavathi Devi, Sakthivel Palaniappan

**Affiliations:** ^1^Department of EIE, Kongu Engineering College, Perundurai, Tamil Nadu 638052, India; ^2^Department of EEE, Velalar College of Engineering and Technology, Erode, Tamil Nadu 638012, India

## Abstract

Convolutional codes are comprehensively used as Forward Error Correction (FEC) codes in digital communication systems. For decoding of convolutional codes at the receiver end, Viterbi decoder is often used to have high priority. This decoder meets the demand of high speed and low power. At present, the design of a competent system in Very Large Scale Integration (VLSI) technology requires these VLSI parameters to be finely defined. The proposed asynchronous method focuses on reducing the power consumption of Viterbi decoder for various constraint lengths using asynchronous modules. The asynchronous designs are based on commonly used Quasi Delay Insensitive (QDI) templates, namely, Precharge Half Buffer (PCHB) and Weak Conditioned Half Buffer (WCHB). The functionality of the proposed asynchronous design is simulated and verified using Tanner Spice (TSPICE) in 0.25 *µ*m, 65 nm, and 180 nm technologies of Taiwan Semiconductor Manufacture Company (TSMC). The simulation result illustrates that the asynchronous design techniques have 25.21% of power reduction compared to synchronous design and work at a speed of 475 MHz.

## 1. Introduction

In today's digital communication systems, convolutional codes are broadly used in channel coding techniques. The main advantage of convolutional coding is that it can be applied to a continuous data stream as well as to blocks of data. Viterbi algorithm [1], due to its high performance, is commonly used for decoding the convolution codes. It is widely used in different communication standards and communication environments like satellite, Wireless Local Area Network (WLAN), and so on. It offers an alternative to block codes for transmission over a noisy channel. Viterbi decoder is the most suitable hardware platform for implementing the Viterbi algorithm. Fast developments in the field of communication in the recent years have created a rising demand for high speed and low power Viterbi decoders with extended battery life, low power dissipation, and low weight. Regardless of the significant progress in the last decade, the problem of power dissipation in the Viterbi decoders still remains challenging and requires further technical solutions. Thus, a flexible, low power, and high speed Viterbi decoder design is a key challenge for future portable and communication devices.

The organization of the paper is as follows.  Section 1 has introduced the scenario in the current digital communication. Sections 2 and 3 present motivation and background on the design styles of asynchronous circuits while Section 4 gives the overview of the Viterbi decoder.  Section 5 details the proposed asynchronous QDI templates and their implementation.  Section 6 reveals the simulation results of asynchronous and synchronous Viterbi decoder and Section 7 concludes the paper.

## 2. Motivation for the Proposed Work

Design of digital circuits is broadly classified into synchronous and asynchronous techniques. Synchronous designs consist of subsystems, which are controlled by one or more clocks that control synchronization and communication between designed blocks. Combinational logic is placed between clocked registers that hold the data of the process. The clock frequency is limited by the delay in the combinational logic and setup time of the register. In fact, the data at the input of the registers may exhibit glitches or hazards as long as they are guaranteed to settle before the clock edge arrives. Asynchronous circuits that do not need a global controlling clock have proved potential benefits in many aspects of digital system design. The clock tree of synchronous circuits consumes significant power and is eliminated in asynchronous ones. Asynchronous methodology uses an event-based handshaking [2] to control synchronization and communication between blocks. Local handshake signals require less power than the clock tree which is used instead of clock signals. Hence asynchronous circuits would consume switching power only when required or when there is a change in inputs. As a result, it is experiencing a significant resurgence of interest and research activity.

Most asynchronous design techniques such as Delay Insensitive (DI), Quasi Delay Insensitive (QDI), speed independent, scalable delay insensitive, bounded delay, and relative timing [3] require some timing assumption or constraints on the wires and components to ensure correct operation. Among the entire timing assumption models, the most commonly used template is the Caltech's QDI templates [4]. QDI is the practical approximation to Delay Insensitive design. It is insensitive to delay of any circuit wires and elements except for some assumptions of isochronic forks. It is more robust and Turing complete and consumes low power. Precharge Half Buffer (PCHB) and Weak Conditioned Half Buffer (WCHB) are the most commonly used QDI templates. The paper proposes the design of an asynchronous low power Viterbi decoder using QDI templates for various constraint lengths. The basic building blocks of asynchronous techniques like Muller C element [4] and capture pass latch [5] are used for designing Survivor Memory Unit (SMU). The internal transistor level design of Viterbi decoder is realized using Differential Cascode Voltage Switch (DCVS) logic because it reduces static power and circuit delay.

## 3. Survey of Existing Works

In the last one decade, researchers have proposed different VLSI techniques for Viterbi decoder. By assuming parallel or pipeline features of hardware resources, Viterbi decoder is designed based on reconfigurable Field Programmable Gate Arrays (FPGA) [6] technology. The conventional maximum likelihood algorithm is redesigned using hardware description language, simulation, and synthesis and implemented (translation, mapping place, and routing), done with FPGA based Electronic Design Automation (EDA) tools.

A Viterbi decoder for the specification of (3, 1, 3) is designed and implemented in HDL [7] with no asynchronous techniques. However, a robust Add Compare Select (ACS) Unit of the Viterbi decoder [8] is designed using asynchronous architecture based on QDI PCFB template to design the internal blocks. Along with the QDI template, Martin synthesis method of directly converting the Communicating Sequential Process (CSP) to transistor level instead of gate level is also used for reducing power dissipation of ACS unit of the decoder.

New, low power memory efficient trace back scheme for high constraint length [9] Viterbi decoder is developed. The buffer based memory bank architecture, due to which the area of the overall proposed trace back is increased, is explored for path merging of SMU.

Analog design of Viterbi decoding [10] for Forward Error Correction (FEC) is used in channel coding for digital communications. Analog is used to reduce the size and power consumption of channel decoders like Viterbi decoders. A differential analog Viterbi decoder architecture is implemented using 32 nm Carbon Nanotube FET (CNTFET) transistors. Increased speed is obtained in the nanotubes, as it holds hefty current and higher driving capacity. The current mode architecture using CNTFETs further reduces the number of transistors, but the analog parameters considered for the design are tedious.

Analysis of different logic styles and their performances in terms of number of transistors, static power, restoring of logic, cascade ability, and robustness are given. From the comparison [11] of the static logic circuits, the Differential Cascode Voltage Switch (DCVS) logic better suits the dual rail encoding in asynchronous QDI.

Variable word length and soft-decision Viterbi decoder [12, 13] which reduces power dissipation in Wireless Local Area Network (LAN) hardware is designed. This is done by considering the dynamic range of the time-varying channel coefficients and adjusting the word length of the decoder to maintain able throughput requirements and low power dissipation. A dynamic voltage scaling technique is applied along with the variable word length to appreciably reduce power consumption in the soft-decision Viterbi decoder.

A two-phase protocol asynchronous Viterbi decoder of radix-4 and *r* = 1/2 [14] based on Muller C-element and double edge triggered *D*-ff is used to ensure the two-phase operation of control signals. The decoder is implemented in Altera cyclone II FPGA. It is reported that the performance of the asynchronous design is better than that of the synchronous part. Similarly, a simple state node method is discussed to reduce the storage [15] resource cost in the design of convolutional code decoders. The state nodes are judged by which the decoder can reach the setup process during each clock cycle.

The low power VLSI architecture asynchronous technique [16, 17] is applied for only BMU and ACS unit. The analysis of power dissipation shows variation in the integrated design of Viterbi decoder. The entire design does not involve the sequence of the state transition graph of PCHB and WCHB buffers. Hence further development of the work concentrating on the complete asynchronous design with buffers and control signals is carried out. The decoder is designed only for the constraint length *K* = 3 while encoder is designed for *K* = 3 to 7 and the results of the encoder are verified for the decoder architecture *K* = 3. It is found that the number of transistor counts is more in asynchronous design for *K* = 3 as the design was simulated on Tanner 7.0 completely in transistors, which leads to the increase in area. This work is reconsidered for designing the decoder for various constraint lengths by incorporating the details of the SMU unit in the present paper. This extended work is carried out on Tanner v13.0 for different technology files.

## 4. Materials and Methods

### 4.1. Implementation of the Viterbi Algorithm in the Proposed Method

**Figure 1:**
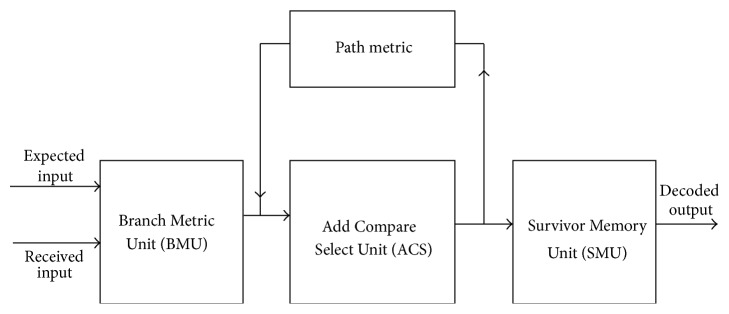
Block diagram of the Viterbi decoder.

The Viterbi algorithm, implemented in hardware, is referred to as the Viterbi decoder. The block diagram of the Viterbi decoder is shown in Figure 1. It is composed of three functional units:

1. Branch Metric Unit (BMU).
2. Path Metric Unit (PMU) or Add Compare Select Unit (ACSU).
3. Survivor Memory Unit (SMU).

### 4.2. Function of BMU

**Figure 2:**
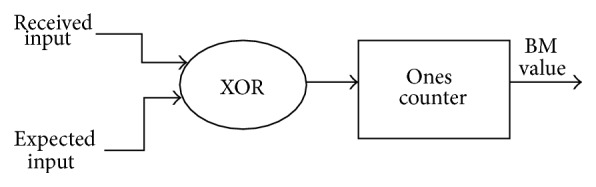
BMU for one state.

BMU, the first unit, consists of XOR gate and counter. The branch metric (BM) computation block compares the received code symbol with the expected code symbol and counts the number of differing bits. The BMU for one state is given in Figure 2.

In hardware implementation, there are two BMUs for a single state, that is, for the upper path (input 0) and lower path (input 1) of the algorithm. There are two inputs for the upper path of the trellis and two for the lower path. One of the two inputs is taken as the received input and the other one as the expected input. The original encoded bit stream is the expected input. The inputs received are obtained by randomly introducing errors in the encoded bit stream.

### 4.3. Working of ACS Unit

**Figure 3:**
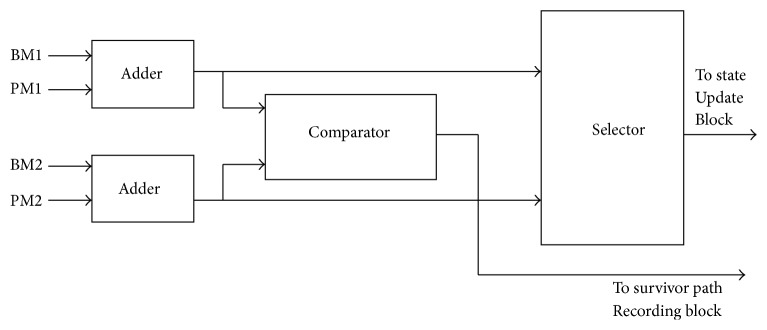
Block diagram of ACS module.

The second unit in the Viterbi decoding is ACSU which is the heart of the process and dictates the performance of the decoder. The ACS operation for each new state in the trellis performs the addition, comparison, and selection of the smallest path metric (PM).  Figure 3 shows the block diagram of the ACS module.

As noted from the above figure, there are two paths for a single state: one path for upper branch and the other for lower branch. The ACSU which adds the BM1 and BM2 to the corresponding PMs, PM1, and PM2, respectively, compares the new PMs and stores the selected PMs in the Path Metric Memory (PMM) in addition to the associated survivor path decisions in SMU.

### 4.4. Function of SMU

To find the survivor path entering each state of the decoder, the BM of a given transition is added to its corresponding PM. This sum (BM + PM) is compared to all the other sums corresponding to all the other transitions entering that state. The transition that has the minimum sum is chosen to be the survivor path.

The third step in the Viterbi decoding is SMU. Three approaches are often used to record survivor branches:

- Trace back (TB) method.
- Register Exchange (RE) method.
- Modified Register Exchange (MRE) method.

RE method assigns a register to each state of BMU. The register records the decoded output sequence along the path starting from the initial state to the final state. The RE technique is acceptable for trellises with only a small number of states, whereas the TB approach is acceptable for trellises with a large number of states. The limitation in TB method is that all the paths of the states have to be traced either forward or backward which involve more transitions and switching activity, thereby increasing latency. RE method is better for implementation on VLSI. This is also the reason why the RE method is updated as MRE method.

In MRE method, there is no need for checking all the paths to identify the minimum value. A pointer keeps track of the minimum PM value. The current decoded bit is appended with the previous decoded values. In the current research, MRE method based SMU concept is used. A pointer is used to monitor the minimum PM and the value is sent to the shift registers directly instead of appending the decoded values. Based on the value of the pointer to the SMU, the corresponding minimum input is chosen. In the minimum input values, if the branch is lower, the decoding bit is 1 and if it is upper branch, the value is taken as 0. The SMU is designed as 4 × 4 shift register using *D*-ff. The length of the shift register depends on the length of the convolution encoder. In SMU, for a constraint length of *K* = 3, there would be 2^*K*−1^ shift registers. The registers that store the minimum path value alone are made active and the remaining registers are kept in idle mode. So, minimum power consumption is obtained in this MRE based SMU method.

## 5. Proposed Asynchronous Architecture and Techniques for Design for Viterbi Decoder 

**Figure 4:**
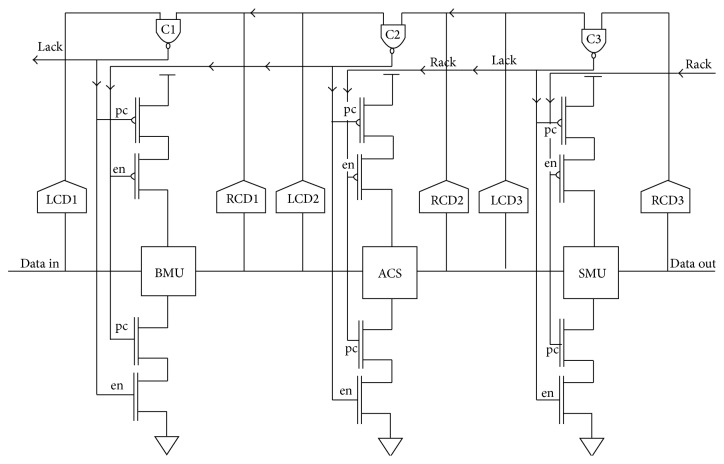
Integrated design of asynchronous Viterbi decoder.

The complete model of Viterbi decoder blocks is implemented using DCVS logic. The decoder is designed for the code rate of 1/2 with the constraint length of *K* = 4 to 7. For ease of considerate, the Viterbi decoder is explained for constraint length *K* = 4. The architecture of the proposed asynchronous Viterbi decoder is presented in Figure 4.

The operation of the asynchronous design is explained with respect to a state transition graph of PCHB template. When the first value is given as input for BMU, LCD1 generates a signal to turn on C1 in order to enable the pc and en signals. The given input value is evaluated by BMU. When the outputs of BMU are validated, completion signal from the RCD1 is sent to the C1 of the BMU stage and LCD2 of the ACS stage. Then ACS unit starts evaluating the data. As soon as the output of ACS is validated, RCD2 generates a completion signal to C2, acknowledgement signal to Lack in the BMU stage, and a request signal to LCD3 unit of SMU. Then BMU unit goes to the precharge phase and SMU is ready for evaluation of current value. Thus, the three stages are executed in a linear pipeline fashion without pipelining registers. The control signals such as se, en, pc, Lo, L1, Ro, R1, and C are designed separately and the circuit is connected in the design wherever necessary.

### 5.1. Branch Metric Unit

The first block of Viterbi decoder is the Branch Metric Unit that is used to compute the branch metric values. It comprises of an XOR gate and a counter. It is used for counting the number of differing bits between the received bits and the expected bits from the channel.

**Figure 5:**
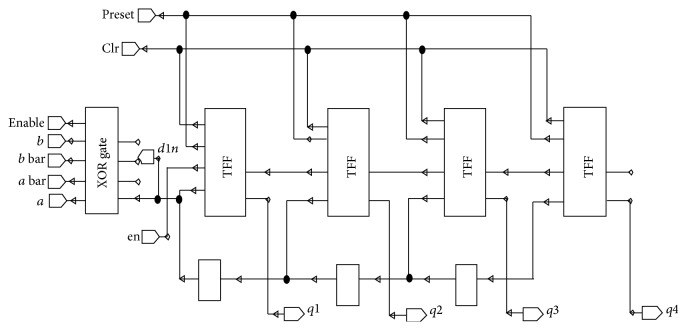
Hardware realization of branch metric computation block.

The hardware realization of the branch metric computation block is shown in Figure 5. PCHB and DCVS logic based design of XOR gate is the first block where *a* and *b* represent true input lines while *a* bar and *b* bar represent the complementary input lines. The output of XOR gate is fed to counter. Since it is based on PCHB, it consists of one more input line “enable” which acts as precharge and evaluation enabled signal that makes the pMOS pull-up transistors be turned on to obtain the output for the design.

The counter in the BMU is constructed by cascading *T* flip-flops. Conventionally, *D* flip-flops are the normal choice for Complementary Metal Oxide Semiconductor (CMOS) circuits. In low power design, design of *D*-ff is more uneasy with whether or not the next state changes; hence *T* flip-flops [18] become the desired choice. The excitation input *T* has the ability to control the switching of the output of a *T* flip-flop. Power dissipation occurs in the clock during both *T* = 0 and *T* = 1. However, it is enviable to reduce the clock power dissipation during *T* = 0. This can be carried out with the excitation function for input *T*.

Thus, the *T* flip-flop is preferred for implementing counting operation. Since it is of asynchronous design instead of clock, enable, preset, and clear signals are used. Preset is the signal that when asserted to “0,” sets the content of storage element to “1” immediately and clear signal and when asserted to “0,” makes the content of storage element to “0” immediately. The output of the flip-flops gives the branch values.

### 5.2. Add Compare and Select Unit

Add Compare Select (ACS) unit is the heart of the process. It consists of 4-bit adder, 5-bit comparator, and the 4-bit selector unit. It computes the lowest path metric value and the decision value. The new path metric value is obtained by adding the previous path metric and the branch metric values.

As the current state is obtained from the earlier stage, the decision value can be represented as one bit. If the decision bit is one, the path metric is selected from the lower state within the two possible states and if the bit is zero, the path metric is selected from the upper state.

**Figure 6:**
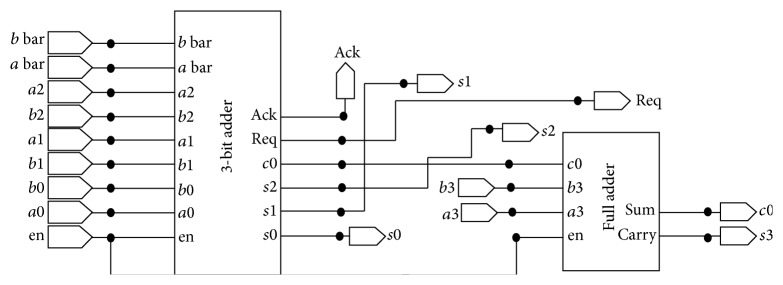
Design of full adder.

In this proposed design, 5-bit asynchronous ripple carry adder is constructed by rippling five 1 bit asynchronous full adders. There are different types of parallel adders. Among them, ripple carry adder is chosen for designing the adder because it requires only less number of transistors and occupies less area. The adder unit is constructed using half adder and full adder. The full adder is constructed by using 3-input XOR gate, 2-input AND gate, and 3-input OR gate. The schematic sketch of full adder is shown in Figure 6.

**Figure 7:**
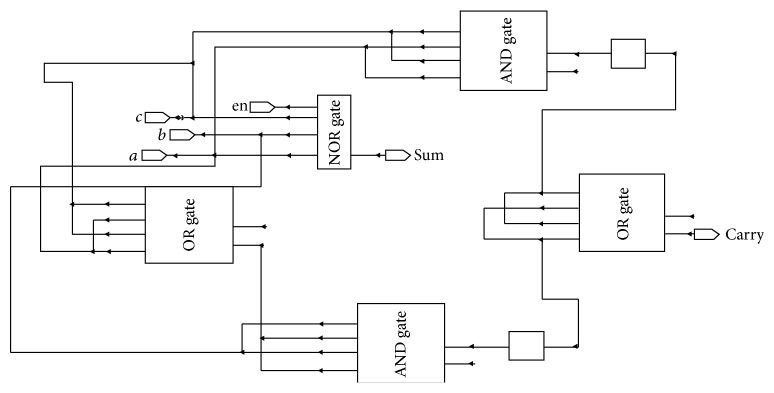
Design of 4-bit ripple carry adder.

**Figure 8:**
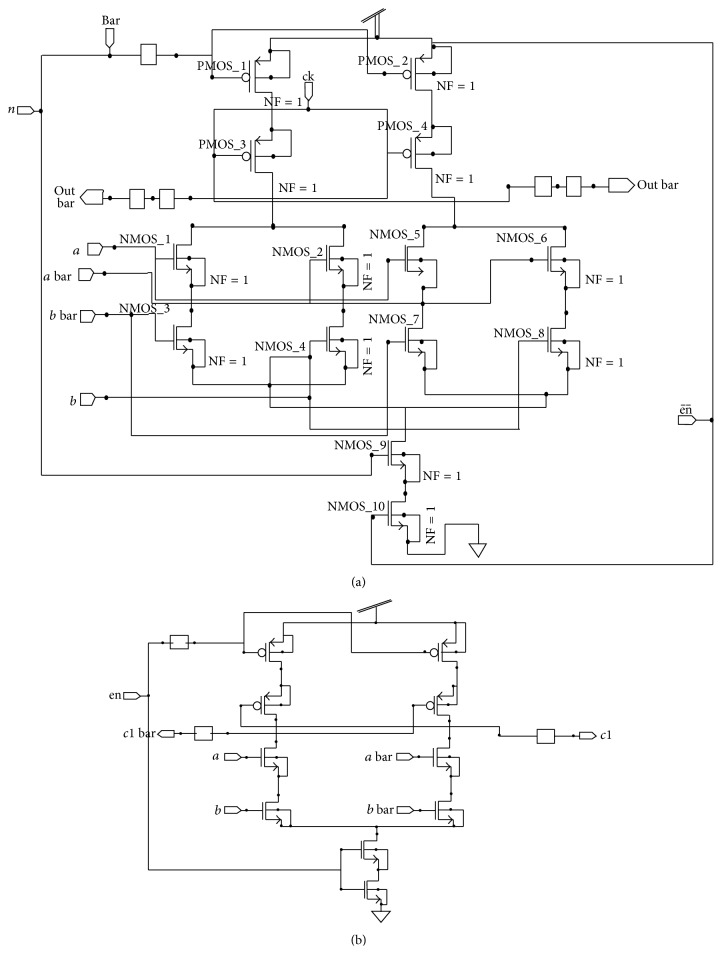
(a) Two-input XOR gates. (b) 2-input AND gate.

Three-bit ripple carry adder is obtained by cascading the half adder and full adder. The carry output of the first half adder is given as one of the inputs of the full adder and similarly for the next adder, carry is propagated as one of the inputs. One set of inputs, namely, *a*  (*a*0, *a*1, *a*2, *a*3), is given from the BMU output. Another set of inputs, namely, *b*  (*b*0, *b*1, *b*2, *b*3), are given from the previous path metric value so that the adder produces the sum output *s*  (*s*0, *s*1, *s*2, *s*3) and carry output *c*0. The diagram for 4-bit ripple carry adder is shown in Figure 7.  Figure 8(a) represents the XOR gate for 3 inputs and Figure 8(b) AND gate for 2 inputs, which are used in the design.

**Figure 9:**
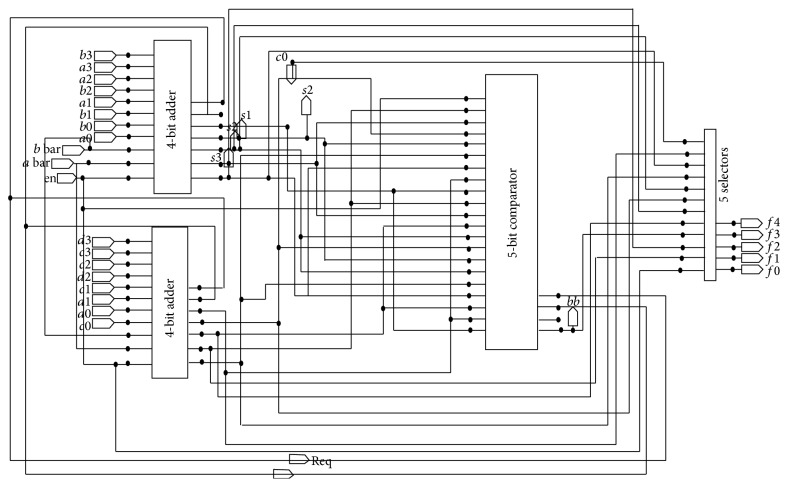
Simulation setup of ACS unit.

The comparator is a combinational circuit which is used to compare the magnitude of 2 inputs. It is used for ACS unit to compare the path metric values and to select the lowest path metric. The output value of the comparator is given to the selector which acts as the selection signal to select the output from the upper and lower adders.  Figure 9 shows the hardware realization of ACS unit. There are request and acknowledgement signals from each block of BMU, ACS, and SMU to coordinate the data operation and completion.

### 5.3. Survivor Memory Unit (SMU)

**Figure 10:**
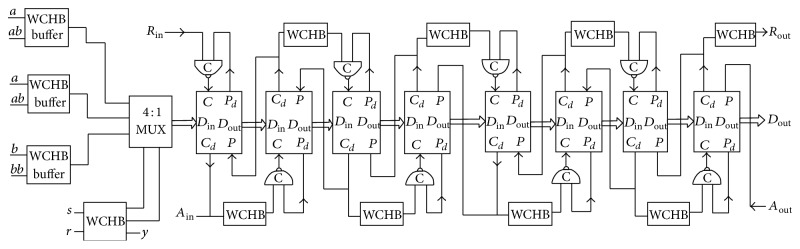
Block diagram of the proposed SMU.

The block diagram of the proposed SMU of Viterbi decoder is shown in Figure 10. Four × four registers are used for each stage with a multiplexer. In the asynchronous architecture, the inputs *a*, *a* bar, *b*, and *b* bar are the inputs to the SMU and the configuration of the registers is serial in serial out fashion. The registers used are capture pass transparent latch which is used to capture and pass the input sequence according to its mode of operation. The design modules Muller C element and WCHB are used instead of clock circuits in order to have low power consumption and to reduce dynamic power dissipation.

The capture pass latch is a type of asynchronous latch which can be used as a data storage element. Sutherland I E 1989 has described the capture pass latch for constructing micro pipeline structures. The latch is transparent between input and output until an event occurs on its capture line. This makes the latch hold the data that are on its input line at that time. During the pass done event, data are passed to the output line. Any change in data input line after pass event has no effect on its output. The latch is used because it has no unwanted switching and low power consumption and is smaller in size.

**Figure 11:**
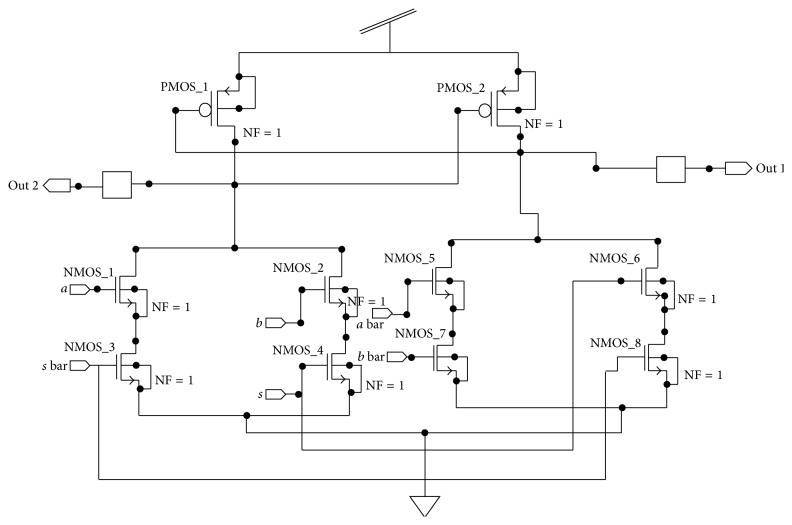
Multiplexer.

In a synchronous circuit, the role of the clock is to define points on time when signals are stable and valid. In between the clock pulses, signals may exhibit hazards and make multiple transitions. In contrast, in asynchronous system, in the absence of clock, all the signals are valid all the time and every transition has significance; consequently, any hazard or race must be avoided. It is used to find the signal transitions in the design. A multiplexer is used for designing SMU to select the input lines. The schematic of multiplexer is shown in Figure 11. In this, *a* and *b* and *a* bar and *b* bar are the input lines while *s* and *s* bar are the selection lines. The output values are represented by* out* and out bar.

## 6. Simulation Results and Analysis

The Viterbi decoder is designed for both synchronous and asynchronous technique by using DCVS logic. The proposed design of Viterbi decoder is simulated in TSPICE for observing the timing behaviour and power consumption for various *K* with the code rate of 1/2.

**Figure 12:**
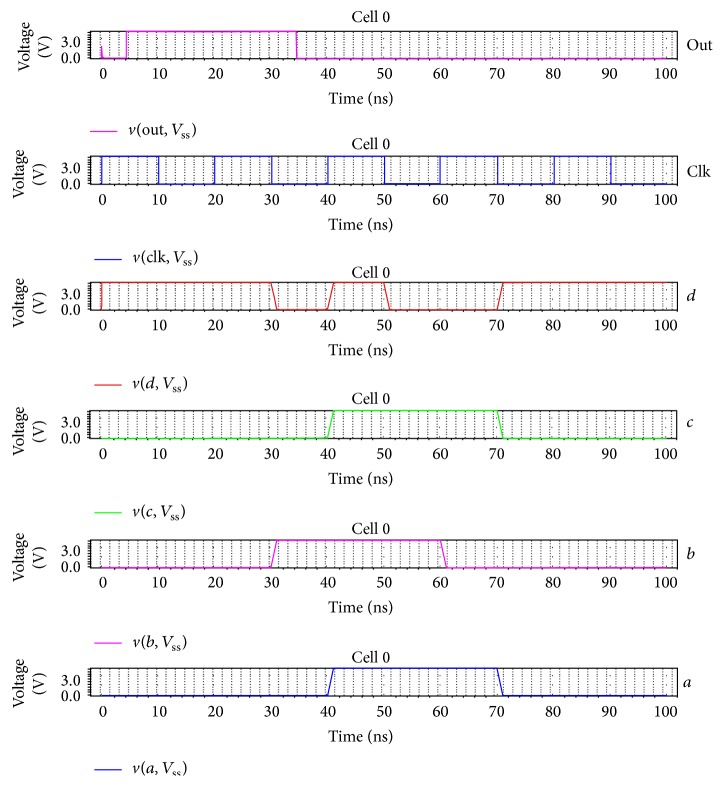
Output waveform of synchronous Viterbi decoder.

**Figure 13:**
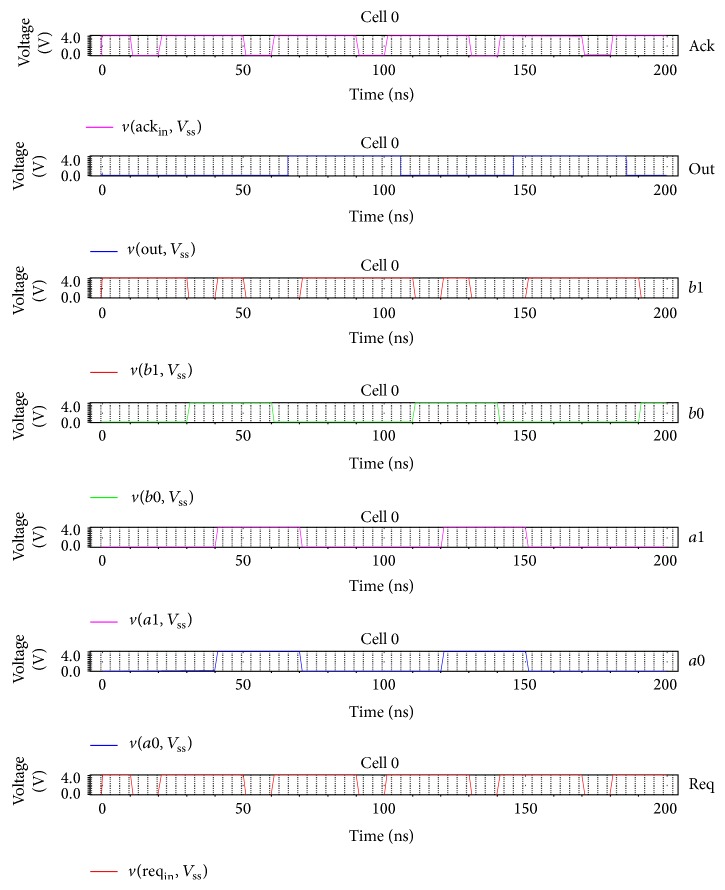
Output waveform of asynchronous Viterbi decoder.

Figure 12 represents the output waveform of the synchronous Viterbi decoder for constraint length *K* = 4 in which the global clock is used for signal transitions. The received sequence input is *a* = *c* = “00 00 11 10” and the expected sequence is *b* = “00 01 11 00” and  *d* = “11 10 10 01.” The output of the synchronous Viterbi decoder is the decoded sequence out = “00 01 10 00.” Similarly, the same set of inputs is given for asynchronous decoder with *a* = *c* = *a*0 = *a*1 and *b* = *b*0 and *d* = *b*1. With the* ack* and* req* signals, the communication between the computing modules is assured. The output of asynchronous decoder is “11 10 10 01” which is shown in Figure 13.

Performance metrics of synchronous Viterbi decoder design for a code rate of 1/2 and for constraint lengths *K* = 4 to 7 for 2^6^ states is shown in Table 1. Comparison of parameters of synchronous Viterbi decoder for various Constraint Lengths shows that synchronous circuit has an average power consumption of 99.245 mW and a frequency of 427 MHz. The results of the synchronous and asynchronous designs are justifiable because of the completion and transition signals in asynchronous design. The completion signal ensures the elimination of additive skew by adding flow control to the circuits. Transition signalling gives an indication of when to begin the data processing without affecting previous output values.

Comparison of parameters of asynchronous Viterbi decoder for various constraint lengths is specified in Table 2. It discusses the gate density, power consumption, and delay for asynchronous Viterbi decoder for various constraint lengths 4 to 7. The average power consumed by the asynchronous Viterbi decoder is 74.03 mW and the frequency of operation is 475 MHz.

From Tables 1 and 2, it is concluded that the asynchronous design is limited by the average case performance rather than the worst case performance in the synchronous design. In addition, the subsystems of the asynchronous Viterbi blocks of *K* = 3 can be replaced for other constraint lengths *K* = 4 to 7 without any clock computation which is not the case in synchronous design. Thus, there is variation of power consumption, delay, and frequency in synchronous Viterbi decoder when compared to asynchronous design.

**Figure 14:**
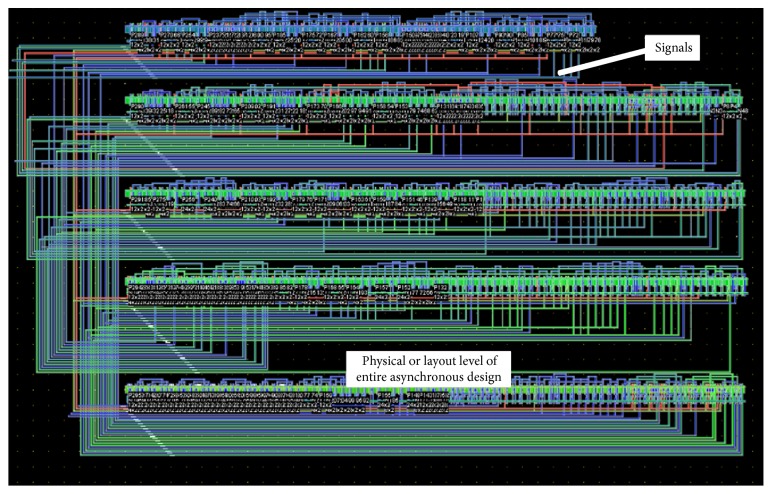
Layout of asynchronous Viterbi decoder.

After the completion of the architectural design and simulation, layout of the asynchronous design is generated from the microwind tool by extracting the spice netlist as given in Figure 14. The layout of the chip has an area of 2700 *μ*m × 2700 *μ*m. Various optimizations are done at this level like slack optimization in ACS and SMU unit of the decoder. All the components in the proposed design are automatically placed and routed by analyzing the critical path in the design. The chip of asynchronous design runs at 1.11 times ahead of its synchronous design because the logic depth is minimum in the critical path due to the DCVSL logic and lack of clock skew.

Table 3 gives the state-of-the-art comparison like technology, number of states, code rate, and maximum speed from Kawokgy and Salama [19]. Various parameters of the comparison show that the proposed asynchronous QDI design of Viterbi decoder outperforms the previous designs with an increase in 60% speed and 10% decrease in power consumption. Normally, the asynchronous circuits that are designed are Delay Insensitive type and each block performs its operation based on the complete signal. Thus, the frequency is higher in the proposed asynchronous system than the other systems, but in the synchronous circuit, though of much less complexity, due to the clock signals, the power consumption is high.

## 7. Conclusion

Viterbi decoders employed in numerous applications such as in LANs, ultra wide band systems, and digital mobile communications are complex in their implementation and they dissipate large power. This paper has presented an improved performance of the Viterbi decoder with asynchronous design for constraint length from *K* = 4 to 7. The Viterbi decoder is also applicable for synchronous design, but with the help of clock signals. The PCHB and WCHB template based asynchronous methodology allows better integration of blocks running at various speeds. The design highlights the usage of the library based cells that are used in the constraint length of *K* = 4 to 7. The simulation is performed using Tanner Spice in TSMC 0.25 *μ*m technology and the design also works for other technology files, namely, 65 nm and 180 nm. As the technology increases, asynchronous designs are less costly for complex designs which can be considered in the long run. From the results, it has been found that the design flow using asynchronous can yield good performance with 25.21% decrease in power consumption compared to the synchronous method.

## Figures and Tables

**Table 1:** Comparison of parameters of synchronous Viterbi decoder for various constraint lengths.

Parameters				
Constraint length (*K*)	Transistor count	Power consumption	Frequency	Delay
4	1520	106.46 mW	330 MHz	3.22 ms
5	2067	114.16	398 MHz	2.51 ms
6	2648	158.09	469 MHz	2.13 ms
7	3152	188.26	512 MHz	1.95 ms

**Table 2:** Comparison of parameters of asynchronous Viterbi decoder for various constraint lengths.

Parameters				
Constraint length (*K*)	Transistor count	Power consumption	Frequency	Delay
4	2116	50.47 mW	433 MHz	3 ms
5	3218	72.54 mW	452 MHz	2.21 ms
6	4320	83.80 mW	488 MHz	2.04 ms
7	5364	89.32 mW	530 MHz	1.88 ms

**Table 3:** Comparison of parameters of Asynchronous Viterbi decoder with the state of art from existing designs [19].

Parameters	Synchronous design	Asynchronous design	Asynchronous design	Asynchronous QDI design
Technology	0.25 um	0.35 um	0.18 um	**180 nm**
No. of states	64	64	64	**64**
Code rate	1/2	1/2	1/2	1/2
Max Speed	200 Mb/s	90 Mb/s	213 Mb/s	**433 Mb/s**
Avg. Power	183 mW	1333 mW	85 mW	**74.03 mW**
